# Infections and perinatal diseases – a comparative overview

**DOI:** 10.1186/1751-0147-49-S1-S10

**Published:** 2007-12-12

**Authors:** Berit Djønne

**Affiliations:** Department of animal health, National Veterinary Institute, Oslo, Norway

**Keywords:** Mastitis, Brucellosis, Definite Host, Bovine Virus Diarrhoea Virus, Leptospirosis

## Introduction

Many different infectious agents, viruses, bacteria, parasites and fungi, can lead to perinatal diseases in animals. Some infectious agents have the genital system as their primary target; they will mainly lead to infertility, abortion, stillbirth or weak newborns. Other agents give a more generalised infection and reproductive problems are only a small part of the picture. This lecture will focus on some important infectious agents that infect the animals during late gestation leading to abortion, stillbirth or foetus anomalies. Infections of the newborn animals the first few days after birth will also be considered.

## Infections during late gestation

### 1. Viral diseases

Many viruses can lead to abortion in different animals, but the reproductive system is seldom their primary target. The lecture will mainly focus on Pestivirus (BVD in cattle, border disease in sheep and classical swine fever), Herpesvirus (infectious bovine rhinotracheitis, equine rhinopneumonitis, canine herpesvirus infections and feline rhinotracheitis), Arterivirus (porcine reproductive and respiratory disease), Retrovirus (feline leukaemia) and Parvovirus (porcint parvovirus, canine distemper and feline distemper).

#### 1.1 Pestivirus

##### Bovine Viral Diarrhoea virus

Bovine Virus Diarrhoea (BVD) and Mucosal Disease (MD) are two variations of the infection. BVD can cause a whole range of disease syndromes in cows. When the virus circulates in the cow, it can reach the growing foetus through the placenta. If a cow that is not immune is exposed to the BVD virus in the first trimester, an early embryonic death or abortion may occur, and if the calf is not expelled from the uterus, it may become mummified. However, if the calf is exposed to the virus between 42 and 125 days of gestation, and if it does not die, it may be born as a "persistently infected" calf. During the second 3 months of gestation, infection may result in abortion, or the calf will be born with birth defects. Generally if a calf is exposed in the uterus during the last trimester, the virus will have no effect on the calf, except that it will be born with antibodies to BVD. Occasionally a late-gestation abortion may result from a BVD virus infection. While immunity in the cow should help to protect the developing foetus, the protection offered is not 100% since there are different strains of BVD virus and only a few virus particles need to reach the foetus to cause an infection. Mucosal Disease results when pestivirus carriers are infected with a second, more aggressive strain of pestivirus. Most carriers develop MD within the first two years after birth. The infection takes a dramatic and eventually fatal course including fever, severe diarrhoea, lesions in the mouth and nose, emaciation and death. There is no treatment for MD. Cases of MD are far less common than the usually mild first-time BVD infections.

##### Border disease

Border disease is an important disease in sheep, caused by infection of the foetus in early pregnancy. Surviving lambs are persistently viremic, and the virus is present in their excretions and secretions, including semen. Ruminants and possibly also pigs can be readily infected by contact with these persistent excretors or with acutely infected sheep. Acute infections in immunocompetent animals usually are transient and subclinical and result in immunity to challenge with homologous but not heterologous strains of virus. The disease is characterised by low birth weight and viability, poor conformation, tremor, and an excessively hairy birth coat in normally smooth-coated breeds. Kids may also be affected, and a similar condition occasionally occurs in calves. The disease has been recognized in most sheep-rearing areas of the world.

##### Classical swine fever

Clinical signs of classical swine fever usually appear 5–10 days after infection (occasionally longer). An individual pig may show one of four types of clinical effect; Peracute (sudden death, especially at the beginning of a farm outbreak), Acute (fever, depression, weakness, anorexia, conjunctivitis, diarrhoea or vomiting, purple discoloration of abdominal skin, or necrosis of the tips of extremities, and neurological signs), Chronic (weight loss, hair loss, dermatitis, discoloration of abdomen or ears) and subclinical. Affected pigs may recover or relapse, depending on the severity of the disease. Reproductive effects is also common; abortions, stillbirths, mummifications and also congenital tremor of piglets.

#### 1.2 Herpes virus

Herpesviruses are divided into three subfamilies and those of veterinary importance are chiefly mucosal pathogens that establish latency within sensory ganglia. Infection with herpesviruses may cause reproductive and neonatal disease in a wide range of species.

##### Infectious Bovine Rhinotracheitis (IBR)

IBR is a serious contagious herpes virus disease of cattle that can cause a variety of different disease syndromes, the most common of which is respiratory disease (pneumonia) and the genital form of the disease complex in both males and females (Infectious pustuloes vulvovaginitis). IBR is a commonly diagnosed viral cause of abortions in cattle. Abortions most commonly occur from 4 months to term, and may occur weeks after the disease has gone through the herd. A cow can also abort if she develops an infectious condition that does not directly affect the foetus.

##### Equine Rhinopneumonitis (Equine Herpesvirus 1)

Equine Rhinopneumonitis is caused by equine herpes virus Type 1 (EHV-1). It occurs in horses of all ages but is more common in horses less than three years old. Sporadic outbreaks come from inhalation of the virus particles. Following a respiratory infection, the virus can cause abortions. Death of the foetus occurs two weeks to four months after exposure to the virus, or during the last three months of pregnancy. Abortion storms have a sudden onset with no additional clinical signs. The virus also cause respiratory problems in foals, and infections near birth can produce weak foals that die within 24 hours.

##### Canine herpesvirus infections (CHV1)

The virus occurs in canine populations throughout the world. It has different effects, depending on the exposed animal. Infection in adults is often inapparent, and latency follows. Reproductive failure manifests as early foetal loss, late-term abortion, stillbirth, or the birth of compromised neonates. Recrudescence of infection in clinically normal animals can occur during periods of physiologic stress. Early neonatal infections result in sepsis and death, with rare survivors suffering permanent neurologic and cardiac deficits.

##### Feline Rhinotracheitis (Feline Herpesvirus 1)

Rhinotracheitis is characterized by respiratory symptoms such as sneezing, nasal discharge, rhinitis, and conjunctivitis. It also affects the reproductive tract and can cause complications during pregnancy. FHV-1 is shed through the discharge from an infected cat's eyes, nose, and mouth. Many cats that are infected with FHV-1 never completely get rid of the virus. These cats are known as latent carriers. Even though they may not show symptoms, they harbour the virus in the nerve cells. Latent carriers spread the infection and are a major source of new infections.

#### 1.3 Arterivirus

##### Porcine reproductive and respiratory disease (PRRS)

The clinical picture of PRRS can vary tremendously from one herd to another, from not recognisable to severe disease. When the virus first enters the breeding herd disease is seen in dry sows, lactating sows, sucking piglets and growers. Clinical signs in dry sows during the first month of infection are inappetence, fever and late term abortions at 1–6% level. These are often the first signs to be noted. Other signs can be discoloration (blueing) of the ears, reproduction problems, coughing and respiratory signs, agalactia and mastitis, mummified piglets (10–15% may die in the last 3–4 weeks of pregnancy), stillbirth (level may increase up to 30%) and birth of very weak piglets. In piglets, more diarrhoea, less viable piglets and increase in respiratory infections can bee seen. Adult animals shed virus for much shorter periods of time (14 days) compared to growing pigs which can excrete for 1–2 months.

#### 1.4 Retrovirus

##### Feline leukaemia

Feline leukaemia virus (FeLV) is a common and important cause of illness and death in cats. Cats that become persistently infected with this virus are at risk of developing severe illnesses such as anaemia and cancer. Between 80 and 90% of infected cats die within three and a half years of being diagnosed as having FeLV. The most common effect of infection is immunosuppression. The virus infects cells of the immune system, killing or damaging them. This leaves the cat vulnerable to a wide variety of other diseases and secondary infections. FeLV infection is often associated with infertility in cats; abortions, stillbirths, and foetal resorption are also more common in FeLV-infected queens. "Fading kitten syndrome" may result from FeLV infection of the foetuses or newborn kittens.

#### 1.5 Parvovirus

##### Porcine parvovirus infection

Porcine Parvovirus Infection (PPV) is the most common and important cause of infectious infertility in pigs. Porcine parvovirus is a fairly tough virus that multiplies normally in the intestine of the pig without causing clinical signs and the virus can live outside the host for many months. It is resistant to most disinfectants. This perhaps explains why it is so widespread and so difficult to remove from the pigs environment. If pregnant sows get infected, an increase in stillbirths associated with mummified piglets, and small litters will be seen. There may be an increase in low birth weight piglets but neonatal deaths are not affected. The acute disease episode often lasts for up to 8 weeks then wanes for 4–6 weeks, followed by some cases of mummified pigs for a further 4–6 weeks. The virus can take up to 4 months to infect all sows in a susceptible previously uninfected herd. Sporadic disease is seen in individual females which are infected for the first time. It is usually confined to gilts. No other signs of ill health in the breeding female or in individual affected animals.

##### Canine distemper

Canine distemper is a contagious, incurable, often fatal, multisystemic viral disease that affects the respiratory, gastrointestinal, and central nervous systems. Distemper is caused by the canine distemper virus. In utero infection of foetuses is rare, but can happen. This can lead to spontaneous abortion, persistent infection in newborn puppies, or the birth of normal looking puppies that rapidly develop symptoms and die within 4 to 6 weeks.

##### Feline panleukopenia

Panleukopenia (feline distemper) is caused by a virus very similar to the one that causes parvovirus disease in dogs. Pregnant cats who become infected with panleukopenia may abort or have stillborn kittens. In some cases, some of the kittens in the litter will be born incoordinated and have tremors, especially of the head. These nervous system changes are caused by the panleukopenia virus affecting the cerebellum, the part of the brain responsible for coordinating muscle movement. Mentally, these cats appear normal. As the kittens grow, they may be able to compensate and lead relatively normal lives.

### 2. Bacterial infections

#### 2.1 Brucellosis

Various *Brucella* species are important zoonotic agents leading to brucellosis in man and animals. Bovine brucellosis is usually caused by *B. abortus*, less frequently by *B. melitensis* and rarely by *B. suis*. Ovine and caprine brucellosis are primarily caused by *B. melitensis*. Porcine brucellosis is caused by *B. suis*, while dogs maimly become infected by *B. canis*.

The Scandinavian countries are declared "officially bovine brucellosis free", but the situation is less favourable in Southern European countries, particularly as far as sheep and goat brucellosis are concerned. Bovine brucellosis is usually asymptomatic in non-pregnant females, but pregnant cows develop a placentitis frequently resulting in abortion in the second half of the gestation. Sometimes the leg joints and the udder are involved in inflammatory processes. Even in the absence of abortion excretion of the infectious organism occurs, for instance via placenta, foetal fluids, vaginal discharge, and milk. Calves are often born immature and weak. Males may develop orchitis and epididymitis. Transmission of the organism during serving and artificial insemination is possible. Ovine and caprine brucellosis is very similar to bovine brucellosis. Sheep is considered to be less susceptible; therefore abortion is less common in ewes. Only sheep can be infected by *B. ovis*. The disease produces inflammation of the epididymis in rams and the placenta in pregnant ewes. A small proportion of infected ewes may abort. If the kidneys are involved, urinary excretion occurs.

#### 2.2 Campylobacteriosis

There are three different bacteria in the genus *Campylobacter* that can lead to veneral infections and abortions. *C. fetus* subsp. *veneralis* gives bovine genital campylobacteriosis, a venereal infection where bulls are carriers. Cows suffer mild metritis, salphingitis and embryonic death with irregular cycles at 28–35 days. The cows will gradually become immune, and clear themselves of the infection. Occasionally cows become carriers. *C. fetus* subsp. *fetus* can give sporadic abortions in cattle. In ewes, both *C. fetus* subsp. *fetus* and *C. jejuni* can cause ovine genital campylobacteriosis. The route of transmission is oral, and carrier animals are the main source of infection. Incubation period is 10–50 days, both bacteria lead to outbreaks of abortions during the last 8 weeks in a flock. A 10–70% abortion rate in susceptible ewes is normal, and the flock immunity lasts for up to three years.

#### 2.3 Leptospirosis

Leptospirosis is a zoonotic disease caused by bacteria from the genus *Leptospira*. The bacteria are mainly spread by animal urine contaminating the environment, and rodents are regarded as a reservoir of the bacteria. The disease can vary, from mild symptoms that are difficult to detect, to an outbreak of fatal cases. All species of mammals, including humans, can be infected, but cattle, dogs, horses and pigs are the most commonly infected animals. Wild animals either do not get sick or develop mild symptoms. In endemic areas, domestic animals often develop chronic disease with few clinical signs of illness. General signs of the disease are fever, anorexia, depression, anemia and dyspnea. More specific signs are abortions, stillbirths and weak newborns, drop in milk yield and mastitis, icterus, hemoglobinuria and conjunctivitis. Mortality in domestic animals does not usually exceed 5% but may reach 20% in small ruminants and even higher in dogs.

#### 2.4 Listeriosis

Listeriosis is a worldwide disease, and affects a wide variety of mammalian and avian species, including man. Encephalitis is the most frequently recognized form of listeriosis of animals. The infection most commonly occurs in adult ruminants that are being fed contaminated silage. *Listeria monocytogenes* and *L. ivanovii* can give abortions in cattle, sheep and goats. The abortions are usually sporadic, and in late gestation. Neither systemic illness nor infertility is associated with the abortions. The bacteria are shed in milk and uterine discharges for some months after infection. *L. monocytogenes* can also give septicaemia in young animals, ocular infections and neural listeriosis, but *L. ivanovii* is only associated with abortions.

#### 2.5 Salmonellosis

The genus Salmonella comprises a single species, *Salmonella enterica*, which has been divided in seven subspecies and about 2000 serotypes. Subspecies I, *S. enterica* subsp. *enterica* contains most of the salmonella serotypes that are animal pathogens. Subspecies IIIb, contains *S. enterica* subsp. *diarizona*, pathogenic for especially sheep. Most Salmonella serotypes are pathogen for all warm blooded animals including humans, making salmonellosis an important zoonosis. Although the salmonellas usually lead to diarrhoea, different serotypes can cause sporadic abortions in cattle, sheep and horses without the animal showing clinical signs of systematic disease. Many abortions may be seen in herds where an outbreak of enteric disease has occurred. Carrier animals and faecal-oral transmission are common. Diagnosis is mainly based on isolation and identification of the agent from placenta and foetuses. *Salmonella* Dublin is especially host adapted to cattle, leading to enteritis, septicaemia, meningitis in calves, abortion, osteomyelitis, joint ill and terminal dry gangrene in calves. *S*. Abortusequi is host adapted to horses, and can lead to abortions in mare, testicular lesions in stallions and septicaemia and polyartheritis in foals. Abortions due to *S*. Abortusequi are rare in Europe. S. enterica subsp. *diarizona* seems to be a special problem in sheep; these variants can cause abortions without any clinical problems in the ewes.

#### 2.6 Tick borne fever

*Anaplasma phagocytophilum* (formerly *Ehrlichia phagocytophila*, *Ehrlichia equi* and *Anaplasma phagocytophila*) is the causative agent of anaplasmosis in humans, horses, sheep, cattle, dogs and cats. The bacteria are transmitted by the tick, *Ixodes ricinus*, therefore the disease is commonly known as tick-borne fever. Infections occur from the bite of an affected arthropod, and the bacteria live inside the mammalian granulocytes and monocytes. The disease is relatively mild, with dullness, fever and immunosuppression. Abortions and stillbirth is quite common in cattle and sheep

#### 2.7 Non-spesific metritis leading to abortions

*Arcanobacterium pyogenes*, *Bacillus* spp, *Streptococcus* spp. and other common bacteria found in the environment can be the cause of sporadic abortions in a dairy herd. These organisms usually get to the placenta and foetus via the cow's circulatory system. While these bacteria may not cause disease symptoms in the cow, the foetus appears to be more susceptible, in large part because of its immature immune system. The resulting growth of bacteria can cause the death of the foetus. Some laboratory data suggests that these bacteria are the most commonly identified cause of bacterial abortions in dairy cattle.

In horses, β hemolyttic Streptococci in group C, *Klebsiella pneumoniae*, *Actinobacillus equuli*, *Pseudomonas aeruginosa*, *Escherichia coli and Salmonella* spp. can give non-specific metritis (dirty mare syndrome) and neonatal septicamiae. Metritis is characterised by vaginal discharges that often become purulent, occasionally accompanied by systematic reaction with fever. If the infection becomes chronic there may be permanent infertility. *Actinobacillus equuli* can infect foals via the umbilical cord, leading to acute septicaemia and sudden death from a few hours after birth to 3 days of age.

### 3. Parasites

#### 3.1 Neospora

*Neospora caninum* is a protozoan parasite of animals, and an important cause of abortions in cattle in many countries. Abortions tend to occur during mid-lactation and it appears that foetuses exposed in early gestation are more likely to abort and that foetuses exposed later are more likely to survive. Although most calves born to infected cows will appear to be clinically normal, there may be some neurological disease that is not readily apparent. Thus far, there is no evidence of disease progression or risk of other diseases in clinically normal, infected calves. Wild or domestic canids are the natural definitive host, and infected animals may shed large numbers of oocysts in their faeces, which may then be ingested by intermediate hosts such as cattle. It is among the most effectively transmitted parasites of cattle and up to 90% of a herd can be infected. Transplacental transmission (from cow to foetus through the placenta) is considered the major route of transmission in cattle. Infection can thus be maintained in dairies even without the definitive host since the infection can pass from cow to calf through many generations.

#### 3.2 Toxoplasmosis

*Toxoplasma gondii* infections in ewes can give abortion and weak lambs. Members of the cat family are the only known definitive hosts for the sexual stages of *T. gondii* and thus are the main reservoirs of infection. Cats become infected with *T. gondii* by carnivorism. After tissue cysts or oocysts are ingested by the cat, viable organisms are released and invade epithelial cells of the small intestine where they undergo an asexual followed by a sexual cycle and then form oocysts, which are excreted. The unsporulated oocyst takes 1 to 5 days after excretion to sporulate (become infective). Although cats shed oocysts for only 1 to 2 weeks, large numbers may be shed. Oocysts can survive in the environment for several months and are remarkably resistant to disinfectants, freezing, and drying, but are killed by heating to 70°C for 10 minutes.

### 4. Mycotic abortions

Fungi can also cause abortions in dairy cattle, most often in the last 2 months of gestation, although they have been observed to occur as early as 60 days. These usually occur during the winter and spring months, since this is when cows are often kept in total confinement and can be exposed to moldy hay or silage. The spores are thought to reach the placenta and foetus through the blood supply of the cow, although the way that they gain access to the circulatory system is not well understood. Fungal abortions tend to occur sporadically although on some occasions a significant percentage (10–20%) of the pregnant animals in a herd may be affected. Mycotic placentitis in horses is also due to an ascending infection that causes a thickened chorioallantois with variable exudate. Causative agents include *Aspergillus*, *Mucor*, and *Candida*. Foetuses aborted in late gestation may be fresh, with evidence of growth retardation. A pale, enlarged liver or dermatitis may be found. Hyphae are found in the placenta, liver, lungs, or stomach contents.

## Infections of the newborn animal

### 1. Diarrhoea

Enteritis in newborn animals is multifactoriel; infectious agents, immune status and management factors are important for development of disease. At birth the intestinal tract is micro-biologically sterile and it has little immunity to disease producing organisms. Organisms begin to colonise the tract quickly after birth, among them potentially pathogenic strains of *E. coli* and *Clostridium perfringens*. Immunity is initially provided by the high levels of antibodies in colostrum (IgG, IgM, IgA). After the colostral antibodies have been absorbed into the blood stream, the immunity is maintained by the antibody (IgA) which is present in milk. IgA is absorbed into the mucous lining of the intestines. It is essential that the newborn animal drinks sufficient colostrum soon after birth to prevent potentially pathogenic organisms multiplying against the intestinal wall and causing diarrhoea. It is also essential that they continue to drink milk regularly after the colostrum has gone so that its intestines continue to be lined by protective antibodies. The antibodies acquired passively from the colostrum and milk can be overwhelmed by large doses of bacteria present in the environment; the higher the number of organisms taken in, the greater the risk of disease. Environmental stress such as chilling also plays a role because it lowers the newborn's resistance. There is thus a delicate balance between the antibody level on the one hand and the weight of infection and stress on the other. Many of the same agents can lead to diarrhoea in all newborn animals; Rotavirus, Coronavirus, *E. coli*, *Clostridium perfringens*, and coccidia. Other agents, as *Salmonella* spp. and worms can give diarrhoea in animals of all ages

#### 1.1 Rotavirus

Rotavirus infections are usually explosive outbreaks of watery diarrhoea, in newborn up to a few weeks old animals. Villus atrophy is a consistent feature with dehydration, malabsorption and wasting. Diarrhoea usually persists for 3–4 days. The infection is usually not severe, and the animals recover if no complications like *E. coli* infections occur. The viruses are widespread, and can persist outside the animal where it is resistant to environmental changes and many disinfectants. Diarrhoea in calves, lambs, foals, piglets, and puppets may be caused by rotavirus. It is estimated that only 10–15% of diarrhoeas in pigs are initiated by a primary rotavirus infection.

#### 1.2 Coronavirus

Coronavirus infections in cattle and sheep resemble rotavirus infections.

##### Transmissible gastroenteritis (TGE)

TGE in pigs can lead to severe watery greenish-gray diarrhoea in pigs. Vomiting is a prominent sign. There will be severe villus atrophy, but without blood in faeces and afebrile animals. Mortality due to dehydration can be 100% in piglets less than seven days old. In non-immune herds, all age groups can be affected.

##### Canine Coronavirus (CCV)

CCV is a highly contagious enteric disease of dogs marked by vomiting and diarrhoea. The virus can replicate in cats, without clinical symptoms. Coronavirus infections are not generally associated with high death rates in puppets.

#### 1.3 Neonatal colibacillosis

There are many different serotypes with different pathogenicity factors among *E. coli*. Variants normally regarded as non-pathogens can cause opportunistic infections in sites of the body such as the mammary gland (mastitis) and uterus (metritis and abortions). *E. coli* strains that cause enteritis has been classified as enteritoxigenic *E. coli* (ETEC), enteropathogenic *E. coli*, enteroinvasive *E. coli* and attaching and effacing *E. coli*. ETEC has fimbrial adhesins F4 (K88), F5 (K99), F6 (987p) and others, and the production of these colonisation factors correlates with enterotoxin production (ST and LT). These strains cause the majority of the neonatal coli diarrhoea.

In calves, lambs and piglets less than one week old, ETEC can give profuse watery diarrhoea and severe dehydration. In a well run herd there should be less than 3% of litters at any one time requiring treatment and piglet mortality from diarrhoea should be less than 0.5%. In severe outbreaks mortality can rise to over 7% and in individual untreated litters up to 100%. *E. coli* infections in piglets are; previously good piglets can be found dead, others huddle together shivering or lie in a corner, the skin around the rectum and tail is wet and there is watery to cream consistency scour with a distinctive smell. As the diarrhoea progresses, the piglets get dehydrated with sunken eyes.

#### 1.4 *Clostridium perfringens*

**Lamb dysenteria** is a clostridial enterotoxemia caused by *C. perfringens* type B. Clinical signs are dysenteria, abdominal pain and continuous bleating. Lambs from newborn to 3 weeks of age get affected.

**Haemorrhagic enterotoxaemia** in piglets is caused by *C. perfringens* type C. Piglets 1–3 days old get severe dysentery, collapse and die. The small intestine is dark red with gas bubbles in mucosa. The lumen of the intestines is often full of bloody fluid. Lambs, foals and calves may develop similar diseases due to *C. perfringens* type C.

#### 1.5 Coccidiosis

Coccidiae are protozoan parasites leading to coccidiosis, one of the most economically devastating diseases in many livestock species. The life cycle of the coccidiae involve a definite host and intermediate hosts. The definite host may develop diarrhoeas, shedding unsporolyted oocysts in the faeces. These oocysts can be taken up by different intermediate hosts where they develop cysts in different tissues. Coccidiosis in the definite host can be especially harmful to recently weaned animals and, occasionally, results in losses in other age groups. It causes watery diarrhoea which is sometimes bloody and can even be a life-threatening problem to an especially young animal. The presence of coccidiae in the intestines of an individual does not mean the animal is actually suffering from coccidiosis as coccidiae are everywhere. These protozoa only cause disease when their numbers become so great that damage is done to the host.

Species of coccidiae and their definite hosts:

*Eimeria* spp. – swine, horses, cattle, sheep, goats

*Toxoplasma gondii* – cat family are the only definite host

*Sarcocystis* spp. – carnivores and perhaps omnivores

*Cryptosporidium* – broad host spectrum including humans,

*C. parvum* are important in calves

*Neospora caninum* – dogs are the only definite host

*Isospora spp*. – dogs, cats, swine (*I. suis*)

### 2. Neonatal septicaemia

In calves, lambs, horses, piglets, pups and kittens less than 1 week old, *E. coli*, *A. pyogenes*, streptococci, staphylococci, and other bacteria can lead to septicaemia, arthritis, and meningitis. The infections are commonly transmitted through the umbilical cord, and the animals can die within few days after the onset of the infection.

Streptococci are common organisms in all animals. They are broadly but not entirely species specific. In horses the β haemolyttic streptococci in group C are important, the main species in pigs is *Streptococcus suis*, in dogs *S. canis* is most important. Streptococci are associated with a variety of conditions including meningitis, septicaemia, polyserositis, arthritis, endocarditis and pneumonia. It has also been isolated from cases of rhinitis and abortion. The pattern and relative importance of the different syndromes vary in different countries.

In horses, *Actinobacillus equuli*, streptococci, *E. coli*, *Salmonella* spp, and *Klebsiella* spp. are most commonly associated with neonatal septicaemias. In cats and dogs, viral septicaemias and respiratory infections are common in all age groups. In cats the fading kitten syndrome is caused by feline leukaemia virus, feline parvovirus, feline coronavirus and bacterial agents, and death of neonates. The fading puppy syndrome is caused by canine herpesvirus, and bacterial septicaemia caused by *E. coli* and streptococci.

